# Retraction: Macrophages Mediate a Switch between Canonical and Non-Canonical Wnt Pathways in Canine Mammary Tumors

**DOI:** 10.1371/journal.pone.0210894

**Published:** 2019-01-10

**Authors:** The PLOS ONE Editors

After publication of this article [1], several concerns were raised about images presented in Figures 1B and 2B.

Similarities were noted between the following images in Figure 1B:

- ccnD1 lanes 1, 2 and wnt5a lanes 2, 1 (horizontally flipped)
- ccnD1 lanes 1–3 and tgfB1r1 lanes 2–4
- tgfB1r1 lane 1 and wnt5a lane 3 (horizontally flipped)
- wnt3 lanes 1–4 and wnt7a lanes 1–4

The following similarities were noted in the upper panels of Figure 2B:

- PKC A/B panel: CMT-U27 cells, lane 1 (ctrl), CMT-U309 cells lane 1 (ctrl; horizontally flipped), and P114 lane 2 (CM, horizontally flipped)
- PKC A/B panel: CMT-U27 lane 2 (CM) and P114 lane 1 (ctrl; horizontally flipped)
- PKC A/B panel: CMT-U309 lane 2 (CM) and P114 lane 3 (+MQ)
- Akt panel: CMT-U27 lanes 1, 2 (ctrl, CM) and CMT-U309 lanes 2, 3 (CM, +MQ), respectively (for these, the bands appear similar but with different intensity suggesting different exposures)
- Akt panel: CMT-U27 lane 3 (+MQ) and P114 lane 1 (ctrl)
- JNK 1/3 panel: CMT-U27 cells, lane 3 (+MQ) and CMT-U309 panel lane 3 (+MQ; horizontally flipped)
- Calmodulin panel: CMT-U309 lane 1 (ctrl) and P114 lane 1 (ctrl; horizontally flipped)

In the lower panels of Figure 2B, it was noted that the band and background in lane 3 of the Lamin a/c CMT-U27 panel (+MQ) appears highly similar to those in lane 1 of the Lamin a/c CMT-U309 panel (ctrl). For these, the bands and background differ in intensity across the panels, suggesting they represent different exposures.

We raised these concerns with the authors, who provided some raw data supporting results in Figures 1 and 2, but noted that the original images supporting the figure panels in question are no longer available. The above similarities call into question the integrity and reliability of the results reported in these figures. The concerns cannot be resolved in light of the data unavailability.

Furthermore, the article is not in compliance with the journal’s Data Availability policy that was in place at the time of the article’s publication.

In light of these concerns, the *PLOS ONE* Editors retract this article.

JM, KM, AH, MB, AM, MG, EH agreed with the retraction. M. Pietrzak, M. Perszko, KR, KP, TM did not respond. MK did not agree with retraction. EM could not be reached.
